# Supplementary material for: Development of Novel Fluorinated Polyphenols as Selective Inhibitors of DYRK1A/B Kinase for Treatment of Neuroinflammatory Diseases including Parkinson’s Disease
Source: Pharmaceuticals (Basel). 2023 Mar 15;16(3):443. doi: 10.3390/ph16030443 (PMC10058583; doi:10.3390/ph16030443)
Supplement: Supplementary file 1 [file pharmaceuticals-16-00443-s001.zip › pharmaceuticals-2266951-supplementary.pdf]

Supplemental Material

Synthesis of (2S,3R)-5,7-bis(benzyloxy)-2-(3,4,5-tris(benzyloxy)phenyl)chroman-3-yl 3,4,5-tris(benzyloxy)-2-fluorobenzoate (**4a**).

Under an N_2 atmosphere, to stirred solution of **3a** (2.2 g, 4.761 mmol, 1.2 eq.) in DCM (10 mL) was added oxalyl chloride (2.1 mL, 19.840 mmol, 5 eq.) and two drop of DMF at 0 °C. The reaction mixture was stirred at RT for 3 h. After this time, the reaction mixture was concentrated under reduced pressure to get acid chloride. The obtained acid chloride was added to a solution of (2S,3R)-5,7-bis(benzyloxy)-2-(3,4,5-tris(benzyloxy)phenyl)chroman-3-ol (3.0 g, 3.968 mmol, 1 eq.), DMAP (1.93 g, 15.870 mmol, 4 eq.) and Et3N (2.2 mL, 15.870 mmol, 4 eq.) in CH2Cl2 (10 mL) at 0 °C. Then the reaction mixture was stirred at RT 16 h. Finally, the reaction was quenched with saturated aqueous NaHCO3 solution (5 mL). The organic layer was separated, and the aqueous layer was extracted with CH2Cl2 (30 mL). The combined organic phase was dried over MgSO4, filtered, and concentrated under reduced pressure. The obtained crude compound was purified by flash column chromatography using EtOAc and hexane as eluent to get **4a** (1.2 g, 70% yield). Analytical Data: ^{1}HNMR (400 MHz, DMSO-d6) δ 7.44–7.22 (m, 40H), 7.04 (d, J = 5.6 Hz, 1H), 6.90 (s, 2H), 6.34 (s, 1H), 6.28 (s, 1H), 5.48 (d, J = 5.2 Hz, 1H), 5.18 (d, J = 7.2 Hz, 1H), 5.12 (s, 2H), 5.06 (s, 2H), 5.01 (s, 4H), 4.94 (s, 6H), 4.87 (s, 2H), 2.98 (dd, J = 5.2 Hz, 1H), 2.81 (dd, J = 7.6 Hz, 1H), ^{19}F NMR (400 MHz, DMSO-d6) δ-133.68. LCMS: (M-H^+): m/Z: 1197.5

Synthesis of (2S,3R)-5,7-dihydroxy-2-(3,4,5-trihydroxyphenyl)chroman-3-yl 2-fluoro-3,4,5-trihydroxybenzoate (**1a**).

To a solution of intermediate **4a** (2.0 g, 1.670 mmol, 1 eq.), in 20 mL of (1:1; THF: MeOH, palladium hydroxide on carbon powder, $Pd(OH)_2$ (20 wt. %, 2.0 g) was added at RT, and the reaction mixture was stirred under hydrogen atmosphere for 16 h. Then the mixture was passed through a pad of celite to remove the catalyst. The filtrate was concentrated under reduced pressure. Obtained crude compound was purified by Prep-HPLC to obtain **1a** as an off-white color solid (0.46 g, 60% yield). Analytical Data: ^{1}HNMR (400 MHz, DMSO-d6): 6.67 (d, J = 6.4 Hz, 1H), 6.24 (s, 2H), 5.90 (d, J = 2.4 Hz, 1H), 5.79 (d, J = 2.4 Hz, 1H), 5.29 (q, J = 5.2 Hz, 1H), 5.03 (d, J = 4.8 Hz, 1H), 2.58 (m, 2H), ^{19}F NMR (400 MHz, DMSO-d6) δ-140.76. LCMS: (M-H^+): m/Z: 475.20.

Synthesis of (2S,3R)-5,7-bis(benzyloxy)-2-(3,4,5-tris(benzyloxy)phenyl)chroman-3-yl 3,4,5-tris(benzyloxy)-2,6-difluorobenzoate (**4b**)

Under an N_2 atmosphere, to a stirred solution of **3b** (0.35 g, 0.735 mmol, 1 eq.) in DCM (8 mL) was added oxalyl chloride (0.31 mL, 7.350 mmol, 10 eq.) in dry DCM (8 mL) and two drops of DMF at 0 °C. The reaction mixture was stirred at RT for 2 h. Then excess oxalyl chloride was concentrated and the residue was dried to get acid chloride. This solution was added dropwise to a solution of **2** (0.44 g, 0.588 mmol, 0.8 eq.), DMAP (0.448 g, 3.675 mmol, 5 eq.) and TEA (0.53 mL, 3.675 mmol, 5 eq.) in DCM (12 mL) at 0 °C. The mixture was stirred at RT for overnight. After this time, saturated $NaHCO_3$ aqueous solution was added. The organic layer was separated and the aqueous layer was extracted with DCM. Combined organic phase was dried over $MgSO_4$ filtered and evaporated. The obtained crude compound was purified by flash column chromatography, eluted with 12% EtOAc in hexane to obtain **4b** as a red solid

(0.109 g, 12% yield). Analytical Data: ^{1}H NMR (400 MHz, DMSO-d6) δ 7.41–7.21 (m, 40H), 6.86 (s, 2H), 6.40 (d, J = 2 Hz, 1H), 6.26 (d, J = 1.6 Hz, 1H), 5.63 (d, J = 5.2 Hz, 1H), 5.17 (s, 4H), 5.11 (s, 2H), 5.06 (s, 4H), 5.02 (d, J = 5.2 Hz, 1H), 4.95 (s, 4H), 4.98 (s, 2H), 2.80 (dd, J = 4.0 Hz, 2H); ^{19}F NMR (400 MHz, DMSO-d6) δ-133.17. LC-MS: (M-H$^+$): m/Z: 1215.47

Synthesis of (2S,3R)-5,7-dihydroxy-2-(3,4,5-trihydroxyphenyl)chroman-3-yl 2,6-difluoro-3,4,5-trihydroxybenzoate (**1b**)

To a solution of **4b** (0.1 g, 0.082 mmol, 1 eq.) in 10 mL of 1:1 THF: MeOH was added palladium hydroxide (20 wt. %, 0.1 g) at RT and the reaction mixture was stirred under hydrogen atmosphere for 16 h. Then the mixture was passed through a pad of celite to remove the catalyst. The filtrate was concentrated under vacuum. Obtained crude compound was purified by Prep-HPLC to obtain **1b** as an off-white color solid (0.026 g, 65% yield). Analytical Data: ^{1}H NMR (400 MHz, DMSO-d6): δ 6.23 (s, 2H), 5.89 (d, J = 2.0 Hz, 1H), 5.77 (d, J = 2.0 Hz, 1H), 5.33 (q, J = 4.8 Hz, 1H), 5.01 (d, J = 4.8 Hz, 1H), 2.58 (dd, J = 4.0 Hz, 2H); ^{19}F NMR (400 MHz, DMSO-d6) δ-145.35. LC-MS: (M-H$^+$): m/Z: 494.95.

Synthesis of (2S,3R)-5,7-bis(benzyloxy)-2-(3,4,5-tris(benzyloxy)phenyl)chroman-3-yl 3,4-bis(benzyloxy)-2-
fluoro-5-methoxybenzoate (**4c**)

To a solution of **3c** (1.13 g, 2.97 mmol, 1.5 eq.) in DCM (12 mL) was added oxalyl chloride (0.9 mL, 9.92 mmol, 5.0 eq.) and 2 drops of dry DMF at 0 °C. The mixture was stirred at RT for 2 h. After the completion of acid-chloride formation, the volatile portion was concentrated from the reaction mixture. The obtained acid chloride was added to a mixture of (2S,3R)-5,7-bis(benzyloxy)-2-(3,4,5-tris(benzyloxy)phenyl)chroman-3-ol (1.5 g, 1.98 mmol, 1.0 eq.), DMAP (0.96 g, 7.93 mmol, 4.0 eq.) and TEA (1.2 mL, 7.93 mmol, 4.0 eq.) in CH_2Cl_2 (20 mL) at 0 °C. The resulting mixture was stirred at RT for 16 h. The reaction mixture was diluted with H_2O (50 mL) and extracted with CH_2Cl_2 (2 X 100 mL). The combined organic layer was washed with brine (50 mL), dried over Na_2SO_4, filtered and concentrated. The residue was purified by flash column chromatography on silica gel (PE/EA = 6/1) to give **4c** (1.51 g, 68% yield) as a white solid. Analytical Data: ^{1}H NMR (400 MHz, CDCl3) δ 7.50-7.20 (m, 35H), 7.00 (d, J = 6.0 Hz, 1H), 6.79 (s, 2H), 6.32 (d, J = 2.0 Hz, 1H), 6.30 (d, J = 2.0 Hz, 1H), 5.50 (q, J = 7.6 Hz, 1H), 5.09 (d, J = 6.0 Hz, 1H), 5.08-4.98 (m, 14H), 3.76 (s, 3H), 3.17 (dd, J = 16.8, 11.2 Hz, 1H), 2.88 (q, J = 8.0, 1H).

Synthesis of (2S,3R)-5,7-dihydroxy-2-(3,4,5-trihydroxyphenyl)chroman-3-yl 2-fluoro-3,4-dihydroxy-5-methoxybenzoate (**1c**)

To a mixture of **4c** (1.50 g, 1.33 mmol, 1.0 eq.) in THF (10 mL) and MeOH (10 mL) was added $Pd(OH)_2/C$ (20 wt. %, 190 mg). The mixture was stirred at room temperature under H_2 atmosphere overnight. The reaction mixture was filtered, and the filtrate was concentrated. The residue was purified by prep-HPLC to give **1c** (0.335 g, 51% yield) as an off-white solid. Analytical Data: ^{1}H NMR (400 MHz, DMSO-d6) δ 9.11 (bs, 7H), 6.60 (d, J = 6.0 Hz, 1H), 6.31 (s, 2H), 5.92 (d, J = 2.4 Hz, 1H), 5.78 (d, J = 2.0 Hz, 1H), 5.18 (q, J = 6.0 Hz, 1H), 4.95 (d, J = 6.4 Hz, 1H), 3.68 (s, 3H), 2.78 (dd, J = 16.4, 5.2 Hz, 1H), 2.88 (dd, J = 16.0, 6.8 Hz, 1H).

Synthesis of (2S,3R)-5,7-bis(benzyloxy)-2-(3,4,5-tris(benzyloxy)phenyl)chroman-3-yl 4,5-bis(benzyloxy)-2-fluoro-3-methoxybenzoate (**4d**)

Under an N_2 atmosphere, to a stirred solution of **3d** (0.265 g, 0.693 mmol, 1 eq.) in DCM (5 mL) was added oxalyl chloride (0.22 mL, 2.665 mmol, 5 eq.) and two drops of DMF at 0 °C. The reaction mixture was stirred at RT for 3 h. The excess oxalyl chloride was removed by distillation, and the residue was dried to give acid chloride. The generated acid chloride was added dropwise to a solution of intermediate **2** (0.203 g, 0.533 mmol, 1 eq.), DMAP (0.325 g, 2.665 mmol, 5 eq.) and TEA (0.36 mL, 2.665 mmol, 5 eq.) in DCM (6 mL) at 0 °C. The mixture was stirred at RT for overnight. After this time, saturated $NaHCO_3$ aqueous solution was added to the reaction mass. Then organic layer was separated, and the aqueous layer was extracted with DCM. Combined organic phases was dried over $MgSO_4$, filtered and concentrated under reduced pressure. The obtained crude compound was purified by flash column chromatography, eluted with 15% EtOAc in hexane, to afford desired compound **4d** as a light yellow solid (0.506 g, 64% yield). Analytical Data: ^{1}H NMR (400 MHz, DMSO-d6) δ 7.43–7.21 (m, 35H), 7.01 (d, J = 9.6 Hz, 1H), 6.89 (s, 2H), 6.42 (s, 1H), 6.27 (s, 1H), 5.51 (d, J = 5.2 Hz, 1H), 5.18 (d, J = 7.2 Hz, 1H), 5.11 (s, 2H), 5.06 (s, 2H), 5.04 (s, 4H), 4.97 (s, 4H), 4.87 (s, 2H), 3.75 (s, 3H), 2.62 (dd, J = 7.6 Hz, 2H). LC-MS: (M-H^+): m/Z: 1121.1.

Synthesis of (2S,3R)-5,7-dihydroxy-2-(3,4,5-trihydroxyphenyl)chroman-3-yl 2-fluoro-4,5-dihydroxy-3-methoxybenzoate (**1d**)

To a solution of **4d** (0.5 g, 0.351 mmol, 1 eq.) in 8 mL of 1:1 THF:MeOH was added palladium hydroxide (20 wt. %, 0.5 g) at RT and was stirred under H_2 atmosphere for 16 h. Then the mixture was passed through a pad of celite to remove the catalyst. The filtrate was concentrated under vacuum. The crude compound was purified by Prep-HPLC to obtain desired compound **1d** as an off-white color solid (0.058 g, 27% yield). Analytical Data: ^{1}H NMR (400 MHz, DMSO-d6): 9.02 (s, 5H), 5.88 (d, J = 6.8 Hz, 1H), 6.25 (s, 2H), 5.91 (d, J = 2.0 Hz, 1H), 5.79 (d, J = 2.4 Hz, 1H), 5.28 (q, J = 5.2 Hz, 1H), 5.01 (d, J = 5.2 Hz, 1H), 3.73 (s, 3H), 2.58 (dd, J = 4 Hz, 2H). LCMS: (M-H^+): m/Z: 490.1.

Synthesis of (2S,3R)-5,7-bis(benzyloxy)-2-(3,4,5-tris(benzyloxy)phenyl)chroman-3-yl 3,4-bis(benzyloxy)-2,6-difluoro-5-methoxybenzoate (**4e**)

Under an N2 atmosphere, to a stirred solution of **3e** (0.6 g, 1.637 mmol, 1 eq.) in DCM (5 mL) was added oxalyl chloride (1.0 mL, 8.168 mmol, 5 eq.) and two drops of DMF at 0 °C. The reaction mixture was stirred at RT for 1 h. After this time, the reaction mixture was concentrated under reduced pressure to get the correspondent acid chloride. Obtained acid chloride was added to a solution of intermediate **2** (1.0 g, 1.637 mmol, 1 eq.), DMAP (0.79 g, 6.548 mmol, 4 eq.) and TEA (0.9 mL, 6.548 mmol, 4 eq.) in DCM (12 mL) at 0 °C. Then the reaction mixture was stirred at RT 16 h. Finally, the reaction was quenched with saturated aqueous $NaHCO_3$ solution (5 mL). The organic layer was separated and the aqueous layer was extracted with DCM (30 mL). Combined organic phase was dried over $MgSO_4$, filtered and concentrated under reduced pressure. Obtained crude compound was purified by flash column chromatography (EtOAc in hexane) to get intermediate **4e** as a yellow solid (0.7 g, 23% yield). Analytical Data: ^{1}H NMR (400 MHz, DMSO-d6) δ 7.44–7.25 (m, 35H), 6.87 (s, 2H), 6.42 (d, J = 2 Hz, 1H), 6.28 (d, J = 1.6 Hz, 1H), 5.48 (d, J = 5.2 Hz, 1H), 5.17 (d, J = 7.2 Hz, 1H), 5.14 (s, 2H), 5.12 (s, 2H), 5.07 (s, 2H), 5.03 (s, 4H), 4.95 (s, 2H), 4.91 (s, 2H), 3.77 (s, 3H), 2.84 (dd, J = 7.6 Hz, 2H); ^{19}F NMR (400 MHz, DMSO-d6) δ-134.46, 134.45, 133.59, 133.58. LCMS: (M-H^+): m/Z: 1139.

Synthesis of (2S,3R)-5,7-dihydroxy-2-(3,4,5-trihydroxyphenyl)chroman-3-yl 2,6-difluoro-3,4-dihydroxy-5-isopropoxybenzoate (**1e**)

To a solution of intermediate **4e** (0.4 g, 0.351 mmol, 1 eq.) in 8 mL of THF:MeOH (1:1) was added palladium hydroxide (20 wt. %, 0.40 g) at RT and the reaction mixture was stirred under hydrogen atmosphere for 16 h. Then the mixture was passed through a pad of celite to remove the catalyst. The filtrate was concentrated under vacuum. Obtained crude compound was purified by Prep-HPLC to obtain the title compound **1e** as an off-white color solid (0.075 g, 29% yield). Analytical Data: ^{1}H NMR (400 MHz, DMSO-d6): 9.05 (bs, 7H), 6.26 (s, 2H), 5.92 (d, J = 2.0 Hz, 1H), 5.80 (d, J = 2.0 Hz, 1H), 5.38 (q, J = 5.2 Hz, 1H), 5.03 (d, J = 5.2 Hz, 1H), 3.73 (s, 3H), 2.56 (t, J = 4 Hz, 2H); ^{19}F NMR (400 MHz, DMSO-d6) δ-141.56, -141.54, -138.98, -138.91. LCMS: (M-H$^+$): m/Z: 508.91

Synthesis of (2S,3R)-5,7-bis(benzyloxy)-2-(3,4,5-tris(benzyloxy)phenyl)chroman-3-yl 3,4-bis(benzyloxy)-5-(difluoromethoxy)benzoate (**4f**)

Under an N_2 atmosphere, to a stirred solution of **3f** (0.71 g, 1.785 mmol, 1.5 eq.) in DCM (6 mL) was added oxalyl chloride (0.5 mL, 5.950 mmol, 5 eq.) and two drops of DMF at 0 °C. The reaction mixture was stirred at RT for 1 h. After this time, the reaction mixture was concentrated under reduced pressure to get acid chloride. The obtained acid chloride was added to a solution of to a solution of intermediate **2** (1.1 g, 1.190 mmol, 1 eq.), DMAP (0.58 g, 4.760 mmol, 4 eq.) and TEA (0.7 mL, 4.760 mmol, 4 eq.) in DCM(5 mL) at 0 °C. Then the reaction mixture was stirred at RT for 16 h. Finally, the reaction was quenched with saturated $NaHCO_3$ aqueous solution (5 mL). The organic layer was separated and the aqueous layer was extracted with DCM (30 mL). The combined organic phase was dried over $MgSO_4$ filtered and concentrated under reduced pressure. The obtained crude compound was purified by flash column chromatography (EtOAc in hexane) to get **4f** as a light yellow solid (1 g, 75% yield). Analytical Data: ^{1}H NMR (400 MHz, DMSO-d6): δ 7.45–7.21 (m, 36H), 7.14 (s, 1H), 6.94 (s, 2H), 6.45 (d, J = 2 Hz, 1H), 6.29 (d, J = 1.6 Hz, 1H), 5.44 (d, J = 5.2 Hz, 1H), 5.22 (d, J = 7.2 Hz, 1H), 5.20 (s, 2H), 5.18 (s, 2H), 5.14 (s, 2H), 5.12 (s, 2H), 5.08 (s, 2H), 4.96 (s, 2H), 4.87 (s, 2H), 3.04 (dd, J = 7.2 Hz, 2H), 2.82 (dd, J = 7.2 Hz, 2H); ^{19}F NMR (400 MHz, DMSO-d6) δ-81.7, -81.38. LCMS: (M-H$^+$): m/z: 1139.53

Synthesis of (2S,3R)-5,7-dihydroxy-2-(3,4,5-trihydroxyphenyl)chroman-3-yl 3-(difluoromethoxy)-4,5-dihydroxybenzoate (**1f**)

To a solution of **4f** (1 g, 0.878 mmol, 1 eq.) in 8 mL THF: MeOH (1:1) was added palladium hydroxide (20 wt. %, 1.0 g) at RT and the reaction mixture was stirred under hydrogen atmosphere for 16 h. Then the mixture was passed through a pad of celite to remove the catalyst. The filtrate was concentrated under vacuum. Obtained crude compound was purified by Prep-HPLC to obtain title compound **1f** as an off-white color solid (0.104 g, 31% yield). Analytical Data: ^{1}H NMR (400 MHz, DMSO-d6): 9.09 (bs, 7H), 6.26 (s, 2H), 5.92 (d, J = 2.0 Hz, 1H), 5.80 (d, J = 2.0 Hz, 1H), 5.38 (q, J = 5.2 Hz, 1H), 5.03 (d, J = 5.2 Hz, 1H), 3.73 (s, 3H), 2.62 (dd, J = 4 Hz, 1H), 2.55 (dd, J = 4 Hz, 1H); ^{19}F NMR (400 MHz, DMSO-d6) δ -81.11. LCMS: (M-H$^+$): m/Z: 508.88.

Synthesis of (2S,3R)-5,7-bis(benzyloxy)-2-(3,4,5-tris(benzyloxy)phenyl)chroman-3-yl 3,4-bis(benzyloxy)-5-
(difluoromethoxy)-2-fluorobenzoate (**4g**)

To a solution of **3g** (0.215 g, 0.51 mmol, 1.0 eq.) in DCM (5 mL) was added $(COCl)_2$ (0.25 mL, 2.57 mmol, 5.0 eq.) and 2 drops of dry DMF at 0 °C. The mixture was stirred at RT for

1 h. After completion of acid-chloride formation, the solvent was evaporated from the reaction mixture and dried under reduced pressure. To this was added a mixture of intermediate **2** (0.39 g, 0.51 mmol, 1.0 eq.), DMAP (0.250 g, 2.05 mmol, 4.0 eq.) and TEA (0.3 mL, 2.05 mmol, 4.0 eq.) in DCM (5 mL) at 0 °C. The resulting mixture was stirred at RT for 16 h. The reaction mixture was diluted with H_2O (30 mL) and extracted with DCM (2 X 50 mL). The combined organic layers were washed with brine (30 mL), dried over Na_2SO_4, filtered and concentrated. The residue was purified by flash column chromatography on silica gel (Petroleum ether/EtOAc, 6/1) to give **4g** (0.21 g, 64% yield) as a white solid. Analytical Data: ^{1}H NMR (400 MHz, CDCl3) δ 7.51-7.30 (m, 33H), δ 7.27-7.21 (m, 3H), 6.77 (s, 2H), 6.34 (t, J = 74.0 Hz, 1H), 6.32 (d, J = 2.0 Hz, 1H), 6.29 (d, J = 2.0 Hz, 1H), 5.51 (q, J = 7.2 Hz, 1H), 5.12-4.98 (m, 15H), 3.14 (dd, J = 10.8, 5.6 Hz, 1H), 3.14 (dd, J = 8.8, 8.0 Hz, 1H).

(2S,3R)-5,7-dihydroxy-2-(3,4,5-trihydroxyphenyl)chroman-3-yl 5-(difluoromethoxy)-2-fluoro-3,4-dihydroxybenzoate (**1g**)

To a mixture of **4g** (0.2 g, 0.17 mmol, 1.0 eq.) in THF (2.5 mL) and MeOH (2.5 mL) was added $Pd(OH)_2/C$ (20 wt. %, 120 mg). The mixture was stirred at room temperature under H_2 atmosphere for overnight. The reaction mixture was passed through a pad of celite and the filtrate was concentrated. The residue was purified by prep-HPLC to give **1g** (40 mg, 44% yield) as a pale pink solid. Analytical Data: ^{1}H NMR (400 MHz, DMSO-d6) δ 8.98 (bs, 7H), 6.97 (t, J = 74.4 Hz, 1H), 6.97 (d, J = 6.4 Hz, 1H), 6.25 (s, 2H), 5.91 (d, J = 2.4 Hz, 1H), 5.78 (d, J = 2.4 Hz, 1H) 5.29 (q, J = 5.2 Hz, 1H), 5.02 (d, J = 5.6 Hz, 1H), 2.62 (d, J = 5.2 Hz, 2H).

Synthesis of (2S,3R)-5,7-bis(benzyloxy)-2-(3,4,5-tris(benzyloxy)phenyl)chroman-3-yl 3,4-bis(benzyloxy)-2,6-difluoro-5-isopropoxybenzoate (**4h**)

Under an N_2 atmosphere, to a stirred solution of **3h** (0.9 g, 2.102 mmol, 1 eq.) in DCM (8 mL) was added oxalyl chloride (0.53 mL, 6.308 mmol, 3 eq.) and two drops of DMF at 0 °C. The reaction mixture was stirred at RT for 1 h. After this time, the reaction mixture was concentrated under reduced pressure to get acid chloride. The obtained acid chloride was added to a solution of **2** (0.079 g, 1.051 mmol, 0.5 eq.), DMAP (1 g, 8.411 mmol, 4 eq.) and TEA (1.1 mL, 8.411 mmol, 4 eq.) in DCM (15 mL) at 0 °C. Then the reaction mixture was stirred at RT 16 h. Finally, the reaction was quenched with saturated aqueous $NaHCO_3$ solution (5 mL). The organic layer was separated, and the aqueous layer was extracted with DCM (30 mL). The combined organic phase was dried over $MgSO_4$, filtered, and concentrated under reduced pressure. The obtained crude compound was purified by flash column chromatography (EtOAc in hexane) to get **4h** as a pale green solid (0.4 g, 32% yield). LCMS: (M-H^+): m/Z: 1167.43

Synthesis of (2S,3R)-5,7-dihydroxy-2-(3,4,5-trihydroxyphenyl)chroman-3-yl 2,6-difluoro-3,4-dihydroxy-5-isopropoxybenzoate (**1h**)

To a solution of **4h** (0.39 g, 0.334 mmol, 1 eq.), in 10 mL of THF:MeOH (1:1) was added palladium hydroxide (20 wt. %, 0.039 g) at RT, and the reaction mixture was stirred under hydrogen atmosphere for 16 h. Then the mixture was passed through a pad of celite to remove the catalyst. The filtrate was concentrated under a vacuum. The obtained crude compound was purified by Prep-HPLC to get **1h** as an off-white color solid (0.094 g, 52% yield). Analytical Data: ^{1}H NMR (400 MHz, DMSO-d6): 9.06 (bs, 7H), 6.24 (s, 2H), 5.90 (d, J = 2.0 Hz, 1H), 5.78 (d, J = 2.4 Hz, 1H), 5.36 (q, J = 4.8 Hz, 1H), 5.02 (d, J = 4.8 Hz, 1H), 4.28-4.22 (s, 1H), 2.60 (d,

J = 4.0 Hz, 2H), 1.19 (s, 3H), 1.17 (s, 3H); ^{19}F NMR (400 MHz, DMSO-d6) δ-139.80, -138.95.
LC-MS: (M-H^{+}): m/Z: 536.96.
